# Infectious Disease in a Warming World: How Weather Influenced West Nile Virus in the United States (2001–2005)

**DOI:** 10.1289/ehp.0800487

**Published:** 2009-03-16

**Authors:** Jonathan E. Soverow, Gregory A. Wellenius, David N. Fisman, Murray A. Mittleman

**Affiliations:** 1 New York University School of Medicine, New York, New York, USA; 2 Cardiovascular Epidemiology Research Unit, Department of Medicine, Beth Israel Deaconess Medical Center, Boston, Massachusetts, USA; 3 Hospital for Sick Children, Toronto, Ontario, Canada; 4 Department of Epidemiology, Harvard School of Public Health, Boston, Massachusetts, USA

**Keywords:** case-crossover study, climate change, global warming, mosquito, vector-borne illness, weather, West Nile virus

## Abstract

**Background:**

The effects of weather on West Nile virus (WNV) mosquito populations in the United States have been widely reported, but few studies assess their overall impact on transmission to humans.

**Objectives:**

We investigated meteorologic conditions associated with reported human WNV cases in the United States.

**Methods:**

We conducted a case–crossover study to assess 16,298 human WNV cases reported to the Centers for Disease Control and Prevention from 2001 to 2005. The primary outcome measures were the incidence rate ratio of disease occurrence associated with mean weekly maximum temperature, cumulative weekly temperature, mean weekly dew point temperature, cumulative weekly precipitation, and the presence of ≥ 1 day of heavy rainfall (≥ 50 mm) during the month prior to symptom onset.

**Results:**

Increasing weekly maximum temperature and weekly cumulative temperature were similarly and significantly associated with a 35–83% higher incidence of reported WNV infection over the next month. An increase in mean weekly dew point temperature was significantly associated with a 9–38% higher incidence over the subsequent 3 weeks. The presence of at least 1 day of heavy rainfall within a week was associated with a 29–66% higher incidence during the same week and over the subsequent 2 weeks. A 20-mm increase in cumulative weekly precipitation was significantly associated with a 4–8% increase in incidence of reported WNV infection over the subsequent 2 weeks.

**Conclusions:**

Warmer temperatures, elevated humidity, and heavy precipitation increased the rate of human WNV infection in the United States independent of season and each others’ effects.

West Nile virus (WNV) is a globally distributed, mosquito-borne flavivirus that caused 3,510 known cases and 109 deaths in the United States in 2007 [Centers for Disease Control and Prevention (CDC) 2007]. The WNV enzootic cycle relies on the vector’s (mosquitoes) interplay with the reservoir (wild birds) and dead-end hosts such as humans, who develop clinical disease after a usual incubation period of 2–6 days (Campbell et al. 2002; Sampathkumar 2003). After its North American debut in New York City in 1999, WNV moved across North America to California, reaching Canada and Central America by 2002 (Hayes and Gubler 2006). The virus’ rapid spread after a drought and during some of the warmest recorded years led to speculation that global climate change aided dispersion (Epstein 2005; National Research Council 2001) and suggests that understanding how weather affects WNV is critical to control efforts.

Like malaria in tropical Africa (Rogers and Randolph 2000; Teklehaimanot et al. 2004) and St. Louis encephalitis in the United States (Defilippo and Epstein 2001; Monath and Tsai 1987; Shaman et al. 2005), increased temperatures influence North American WNV distribution. Above-average temperatures correlated with WNV’s spread into western states and with county-level mosquito infectivity (Reisen et al. 2006), high 2002 northeastern metropolitan case loads (El Adlouni et al. 2007), and the transfer of virus from a secondary to a primary bridge vector (Kunkel et al. 2006). Higher temperatures also have important effects on mosquitoes that carry WNV, increasing viral load and shortening the extrinsic incubation period (EIP) under laboratory conditions, and, like humidity, accelerating blood-feeding and reproductive activity in the field (Dohm et al. 2002; Reisen et al. 2006; Shaman and Day 2007).

In contrast, the effects of precipitation on WNV and other U.S. mosquito-borne disease transmission remain controversial (Degroote et al. 2008; Shaman and Day 2007). Broad regional trends suggest that prior drought contributed to the initial U.S. WNV outbreak (Degroote et al 2008; Hubalek 2000), but subsequent research has been inconsistent, showing both positive and negative associations with rainfall and WNV and similar mosquito-borne diseases (Hubalek 2000; Landesman et al. 2007; Shaman et al. 2003). WNV vector populations have increased (Landesman et al. 2007) and decreased (Degaetano 2004) after elevated precipitation, depending on location and calendar month.

The Intergovernmental Panel on Climate Change (IPCC 2007) projects that climatic and weather conditions in North America in the coming decades are likely to include warmer temperatures, shorter winters, increased proportion of precipitation falling as rain rather than snow, and increased frequency of heavy rainfalls and other extreme weather events. If temperature and precipitation are influential in determining WNV infection risk, such changes would be likely to increase the burden of this disease in coming decades. Associations between meteorologic variables and risk of WNV case occurrence have not been systematically evaluated across geographically diverse regions. Accordingly, we studied the effects of ambient temperature, humidity, and precipitation on the incidence of WNV infection among 16,298 cases reported to the CDC between 2001 and 2005 in 17 U.S. states.

## Methods

### Case data

We obtained dates of symptom onset, age, and county location for human cases during 2001–2005 from the CDC using their case definition (CDC 2004). A case must meet one or more clinical criteria (altered mental status, neurologic dysfunction, or pleocytosis) plus one or more laboratory criteria (for a confirmed case: 4-fold change in serum antibody titer, isolation of virus, virus-specific IgM in the cerebrospinal fluid, or virus-specific IgM plus demonstration of virus-specific IgG antibodies at a later date; for probable case: stable but elevated virus-specific serum antibody titer or virus-specific serum IgM antibodies). Because data on all states were not available to us, we obtained data from 17 states with large case numbers to achieve sufficient power, study efficiency, and geographic diversity. The states included were Illinois, Pennsylvania, Michigan, Indiana, Ohio, South Dakota, North Dakota, Nebraska, Montana, Wyoming, Idaho, Colorado, Texas, Louisiana, Arizona, California, and New Mexico.

### Meteorologic data

We obtained daily data on ambient temperature, dew point temperature, and precipitation from the National Oceanic and Atmospheric Administration (NOAA) National Climatic Data Center (NOAA 2008). Only data from NOAA-designated first-order weather stations were obtained to ensure data completeness, consistency, and accuracy.

A degree-day is a measure of cumulative daily temperature within a temperature band. We calculated degree-days using the single-sine method (Allen 1976), which models daily temperature on a sine wave using minimum and maximum values to determine the day’s accumulated degrees above our threshold of 14°C. Thus, if the temperature in a given day remained at 15°C, 1 degree-day would be recorded. The single-sine approach has been validated against other estimation methods (Roltsch et al. 1999) and is a common and easily replicable variable. We chose the lower threshold of 14°C based on laboratory evidence demonstrating this viral transmission limit in mosquitoes (Reisen et al. 2006). We modeled degree-days using a lower but not an upper temperature threshold because of uncertainty surrounding the ultimate effects of extreme heat on both viral loads and mosquito survival.

We performed a geographic analysis to match meteorologic data with individual cases. Specifically, we created a map of all case-positive counties and superimposed the location of all U.S. first-order weather stations based on their latitude and longitude. In counties with multiple weather stations, we calculated a daily average across all stations. Counties lacking weather stations (or months during which weather stations were not operational) were paired with the closest station to the county’s geometric centroid using ArcGIS 9.2 (ESRI, Redlands, CA, USA).

### Study design and analysis

We used the case–crossover study design to evaluate the association between meteorologic variables and WNV case occurrence. The case– crossover study design was specifically developed to study the effect of transient exposures on the risk of acute events (Maclure and Mittleman 2000). In this design, each subject’s exposure prior to a case-defining event (case period) is compared with his or her own exposure experience during one or more control periods when the subject did not become a case. Thus, each case serves as his or her own control. Because there is perfect matching on all measured and unmeasured time-invariant subject characteristics, there can be no confounding by risk factors that are stable over time within each subject. For each case, we selected one control period from the same day of the week 4 weeks before or after the case date, using a random, bidirectional selection procedure previously described by Navidi (1998). Navidi (1998) showed that sampling control periods only from before the index event leads to bias in the presence of time trends in exposure or potential confounders, as would be expected in studies of atmospheric phenomena. Lumley and Levy (2000) showed that with a rare event, such as WNV infection, any bias from sampling referents after the index event is small and importantly, much smaller than the bias from time trends in exposure or potential confounders. Control periods were chosen 4 weeks before or after the index event to effectively control for temporal trends but without overlap between the case and control exposure periods.

During the month prior to symptom onset, increasing temperatures accelerate the EIP and expand mosquito viral load, increasing the likelihood of viral spread (Reisen et al. 2006). We chose *a priori* to use maximum daily temperature as the primary temperature measure because previous studies demonstrated an association with maximum values (Kunkel et al. 2006). However, in our sample, maximum daily temperature correlated highly with both mean daily (*r* = 0.95) and minimum daily temperatures (*r* = 0.73). We also modeled cumulative temperature using the single-sine method (Allen 1976) to evaluate the potential for threshold effects of a lower temperature boundary. We hypothesized that although elevated precipitation may increase mosquito populations, torrential downpours may decrease them. Accordingly, we assessed the effects of cumulative weekly precipitation as a continuous variable and having ≥ 1 day of heavy (> 50 mm) (Shaman and Day 2007) precipitation in a week.

The analysis of case–crossover data is an application of standard methods for stratified data analysis. We performed conditional logistic regression, stratifying on each case, to obtain exposure odds ratios (ORs) as estimates of incidence rate ratios and 95% confidence intervals (CIs) associated with meteorologic variables. Using a distributed lag model (Schwartz 2000), both univariate (each meteorologic variable considered separately) and multivariable-adjusted (variables entered simultaneously) analyses were performed using SAS software (SAS Institute Inc., Cary, NC, USA). Results from our primary analyses are presented for a 5°C increase in maximum temperature or dew point and for a 20-mm increase in cumulative weekly precipitation. In sensitivity analyses, we used cumulative weekly temperature or mean weekly mean temperature instead of mean weekly maximum temperature and compared the effects by scaling regression estimates to the interquartile range of each metric among the control periods in our sample.

## Results

**Figure 1:**
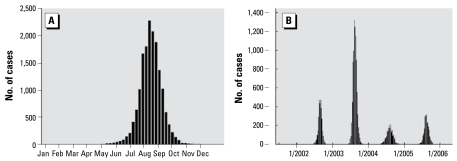
Number of human WNV cases by week (*A*) and by week for each year (*B*) in the study sample.

We assessed 16,298 human WNV cases during 2001–2005 across 17 states. Cases ranged from < 1 to 99 years of age, with a median age of 49 years, and spanned the entire calendar year (Figure 1). Three hundred fifty-one first-order weather stations supplied data for case occurrence in 667 counties at a median distance of 13.8 miles from the respective county center.

### Temperature and humidity

**Figure 2:**
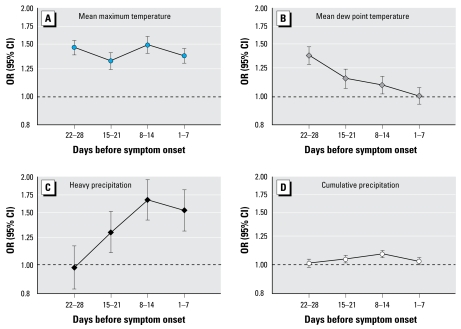
OR and 95% CIs of reported WNV infection for a 5°C increase in maximum temperature (*A*) or dew point temperature (*B*), for the presence of ≥ 1 day with > 50 mm rainfall (*C*), or for a 20-mm increase in cumulative weekly precipitation (*D*).

In a model controlling for dew point temperature and precipitation, mean weekly maximum temperature was positively and significantly associated with the incidence of reported WNV infection during the same week and in the subsequent 3 weeks (Figure 2A). Specifically, a 5°C increase in mean maximum weekly temperature was associated with a statistically significant 32–50% higher incidence of reported WNV infection. Similar results were obtained when a cumulative weekly temperature > 14°C or a mean weekly mean temperature were considered instead of mean weekly maximum temperature (Table 1). Limiting the analysis to cases occurring June–August of each year did not materially change the results.

In a model controlling for mean weekly maximum temperature and cumulative weekly precipitation, mean weekly dew point temperature was significantly associated with the incidence of reported WNV infection 1–3 weeks later, but not during the same week (Figure 2B).

### Precipitation

In a model adjusting for mean weekly maximum temperature and mean dew point temperature, a 20-mm increase in cumulative weekly precipitation was associated with a 4–8% increase in incidence of reported WNV infection 1–2 weeks later (Figure 2D). Cumulative weekly precipitation was not associated with the rate of reported WNV infection on the same week or 3 weeks later.

One or more days per week of heavy precipitation (defined as ≥ 50 mm in a single day) was associated with a 33% (95% CI, 15–54) higher incidence of reported WNV infection during the same week, and the incidence remained elevated in the subsequent 2 weeks (Figure 2C). Lowering the threshold of heavy precipitation to ≥ 40 mm or ≥ 30 mm progressively attenuated the association (Table 2). Limiting the analysis to cases occurring June–August of each year did not materially change the results.

## Discussion

In this study we examined the influence of temperature, humidity, and precipitation on the incidence of reported WNV infections among 16,298 reported human cases. We found positive associations with increasing temperature over each of the 4 weeks prior to symptom onset. In addition, heavy but not low levels of rainfall were significantly and positively associated with the incidence of reported WNV infections over the same week, while both were significantly and positively associated with WNV infections over the subsequent 2 weeks. Although insignificant during the case week, elevated weekly dew point temperature progressively increased the incidence of reported WNV infections over the ensuing 3 weeks.

Our results, which were maximal after a lag of 1–2 weeks for both temperature and rainfall, map onto the period when they might best accelerate reproductive activity and the probability of an infectious bite. Time from oviposition to adulthood in *Culex* is approximately 8–12 days, and the EIP under laboratory conditions ranges from 4 to 12 days at 30°C and > 28 days at 18°C (Dohm et al. 2002). The typical incubation period in humans is 2–6 days, stretching back as far as 14 days (Campbell et al. 2002; Petersen et al. 2003).

We assessed the effects of ambient temperature using three metrics: mean weekly maximum temperature, mean weekly mean temperature, and weekly cumulative temperature (i.e., accumulated weekly degree-days). All of these measures of temperature yielded a similar pattern of incidence rate ratios during each of the 4 weeks, with the maximum rate occurring at a lag of 1 week. On days when the temperature did not drop below our minimum threshold of 14°C, the degree-days calculation approximates the mean temperature above the threshold. The fact that the association was not universally stronger with accumulated degree-days than with mean weekly maximum temperature suggests that conditions below the specific threshold of 14°C did not significantly attenuate the rate of WNV transmission, as would be predicted based on experimental data in the laboratory.

The strongest effects of both light and heavy precipitation appear to transpire 1–2 weeks after the rainfall, during a period when they might expand mosquito populations and influence mosquito host-seeking. We found that the definition of “heavy” precipitation significantly influenced the results, with the strongest associations observed using a definition of ≥ 50 mm precipitation in a single day. This finding may imply that large, single-day rainfalls influence transmission in a distinct manner from cumulative precipitation. In turn, the fact that heavy but not light rainfall was positively associated with incidence over the same week suggests that a downpour may increase human–mosquito interaction and the likelihood of an infectious bite. Prior research suggests that heavy rainfall can stimulate episodes of disease transmission by increasing near-surface humidity, which stimulates mosquitoes to oviposit and seek hosts (Shaman and Day 2007). However, adjusting for temperature and rainfall, weekly dew point temperature was not significantly associated with the incidence of reported WNV during the same week. Our results depict humidity’s influence increasing with time, arguing that the mechanism of its effects is through mosquito breeding rather than biting, given the typical incubation period of 2–6 days.

This study has important strengths and limitations that merit discussion. First, we had data only on the county of residence of each case, leading to possible misclassification of exposure due to heterogeneity in weather conditions within counties. Errors in reported dates of symptom onset may also contribute to exposure misclassification. However, both of these sources would be expected to bias our effect estimates toward the null. Second, the relationship between meteorologic conditions and vectorborne disease risk are enormously complex (Rogers and Randolph 2000), invoking mosquito feeding preference (Kilpatrick et al. 2006), terrain and vegetation index (Ruiz et al. 2007), and bird migration patterns (LaDeau et al. 2007; Samuel and Diamond 2006). It was our intention to assess broad trends using easily measured exposures eventually applicable to public health planning. On the other hand, this study has several important strengths including large sample size, geographic diversity, and the use of the case–crossover design, which controls for confounders such as age, immune status, season, and location that remain relatively stable over short time periods.

Because the impacts of major meteorologic phenomenon such as global warming on temperature and precipitation are varied and occur over long time periods, the effects of climate shifts could not be evaluated in this study, which is designed to assess the effect of meteorologic variations on a shorter time scale. Still, our results carry implications for future studies of major climatic trends. Although many factors influence WNV incidence, this study indicates that increasing temperature, humidity, and rainfall across the United States independently and transiently elevates WNV incidence over the subsequent month regardless of season and location. This knowledge may complement traditional surveillance techniques and suggests, if weather trends continue, that the human impact of this and related diseases may increase in coming decades.

## Conclusion

Warmer temperatures, elevated humidity, and heavy precipitation increased the relative rate of human WNV infection in the United States independent of season and each others’ effects.

## Figures and Tables

**Table 1:** OR (95% CI) of reported WNV infection for different metrics of ambient temperature.

Days before symptom onset	Mean weekly maximum temperature	Weekly cumulative temperature	Mean weekly mean temperature
1–7	1.53 (1.41–1.65)	1.35 (1.24–1.47)	1.52 (1.39–1.67)
8–14	1.72 (1.57–1.87)	1.83 (1.66–2.01)	1.73 (1.55–1.92)
15–21	1.45 (1.33–1.58)	1.45 (1.32–1.59)	1.62 (1.45–1.79)
22–28	1.67 (1.55–1.80)	1.62 (1.50–1.76)	1.87 (1.71–2.05)

Rate ratios are for an interquartile range increase in mean weekly maximum temperature (6.7°C), weekly cumulative temperature > 14°C (36.6 degree-days), and mean weekly mean temperature (6.2°C). Results are from multivariable models controlling for mean weekly dew point temperature and weekly cumulative precipitation.

**Table 2:** OR (95% CI) of reported WNV infection after weeks with ≥ 1 day of heavy precipitation for different definitions of “heavy.”

	Precipitation		
Days before symptom onset	≥ 50 mm	≥ 40 mm	≥ 30 mm
1–7	1.53 (1.30–1.80)	1.38 (1.21–1.57)	1.18 (1.06–1.31)
8–14	1.66 (1.42–1.94)	1.31 (1.15–1.49)	1.26 (1.13–1.40)
15–21	1.29 (1.10–1.51)	1.28 (1.12–1.45)	1.19 (1.06–1.32)
22–28	0.98 (0.83–1.16)	0.87 (0.76–0.99)	0.86 (0.77–0.96)

Results are for a multivariate analysis controlling for mean weekly maximum temperature and mean weekly dew point temperature.
